# Discovery of Novel Small Molecule Anti-HCV Agents via the CypA Inhibitory Mechanism Using *O*-Acylation-Directed Lead Optimization

**DOI:** 10.3390/molecules200610342

**Published:** 2015-06-04

**Authors:** Wenzhong Yan, Jie Qing, Hanbing Mei, Fei Mao, Jin Huang, Jin Zhu, Hualiang Jiang, Lei Liu, Linqi Zhang, Jian Li

**Affiliations:** 1 Shanghai Key Laboratory of New Drug Design, School of Pharmacy, East China University of Science and Technology, 130 Mei Long Road, Shanghai 200237, China; E-Mails: 15901872753@163.com (W.Y.); xiuzhifumhb@126.com (H.M.); maofei@ecust.edu.cn (F.M.); huangjin@ecust.edu.cn (J.H.); jinz@ecust.edu.cn (J.Z.); hljiang@ecust.edu.cn (H.J.); 2 Tsinghua-Peking Center for Life Sciences, Key Laboratory of Bioorganic Phosphorus Chemistry & Chemical Biology (Ministry of Education), Department of Chemistry, Tsinghua University, Beijing 100084, China; E-Mail: qing282420@163.com; 3 School of Medicine, Tsinghua University, Beijing 100084, China; 4 Drug Discovery and Design Center, Shanghai Institute of Materia Medica, Chinese Academy of Sciences, 555 Zu Chong Zhi Road, Shanghai 201203, China

**Keywords:** CypA, small molecule inhibitor, anti-HCV, *O*-acylation

## Abstract

In this work, the relationship between cyclophilin A (CypA) and HCV prompted us to screen a series of small molecule CypA inhibitors which were previously reported by our group. Among them, compound **1**, discovered as a non-immunosuppressive anti-HCV agent with an EC_50_ value of 0.67 μM in a virus assay, was selected for further study. Subsequent chemical modification by *O*-acylation led to a novel class of molecules, among which compound **25** demonstrated the most potent anti-HCV activity in the virus assay (EC_50_ = 0.19 μM), but low cytotoxicity and hERG cardiac toxicity. The following studies (a solution stability assay and a simple pharmacokinetic test together with a CypA enzyme inhibition assay) preliminarily indicated that **25** was a prodrug of **1**. To the best of our knowledge, **25** is probably the most potent currently reported small molecule anti-HCV agent acting via the CypA inhibitory mechanism. Consequently, our study has provided a new potential small molecule for curing HCV infection.

## 1. Introduction

Infection with hepatitis C virus (HCV) is well recognized as a worldwide health issue that chronically affects more than 170 million people [1,2,3]. Therefore hepatitis C has been one of the most active areas for antiviral drug development over the past two decades [4,5]. Currently, there are more than 20 directly-acting antivirals (DAAs) targeting the HCV NS3 protease, NS5B polymerase and NS5A protein in advanced clinical trials [6]. However, these DAAs have to be used in combination clinically to avoid resistance, because HCV, an RNA virus, possesses high replication and mutation rates [6]. Thus, a strategy targeting host factors essential for viral replication such as cyclophilins (Cyps) may create alternative kinds of anti-HCV agents for clinical application alone or in combination.

Cyps are a family of proteins found in vertebrates and other organisms that are proved to be identical to peptidyl-prolyl *cis-trans* isomerase (PPIase) [7]. Via its PPIase activity, Cyps play a crucial role in numerous cellular processes, including transcriptional regulation, immune response, protein secretion, and mitochondrial function [8,9]. Cyclophilin A (CypA), the most critical member of the Cyp family, has been extensively researched. Except for the functions mentioned above, CypA also plays a critical part in the replication of various kinds of viruses, such as human immunodeficiency virus type 1 (HIV-1), influenza virus, HCV, vesicular stomatitis virus (VSV), vaccinia virus and human papilloma virus (HPV) [10,11]. On account of the importance of CypA in the regulation of numerous biological processes, significant efforts have been made in the discovery of CypA inhibitors in the past decade [12,13,14,15,16,17].

**Figure 1:**
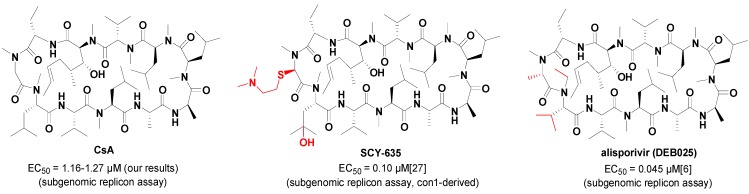
Chemical structures and anti-HCV activities of CsA, SCY-635 and alisporivir.

In recent years, the relationship between HCV and CypA has been demonstrated more and more clearly [18,19,20,21]. Abundant research has shown that CypA is an important host factor for HCV proliferation as the PPIase activity of the hydrophobic gorge area in CypA plays a critical role in the HCV RNA proliferation and protein secretion [20,22]. This enzyme can enhance the rate of folding/unfolding of proteins via its PPIase activity, and thereby guarantee the correct assembly and functions of the HCV replicative complex [8]. In the latest studies HCV NS5A protein was proved to be the main ligand of CypA, which was essential for the prolyl peptide isomerization of NS5A in the process of HCV proliferation, by various methods, including nuclear magnetic resonance (NMR), isothermal titration calorimetry (ITC), and surface plasmon resonance (SPR) [23,24,25]. Thus, CypA PPIase inhibitors could hinder virus proliferation by disrupting the interaction between CypA and NS5A [23,24,25]. Currently, all reported CypA inhibitors for anti-HCV in clinical studies are cyclosporin A (CsA) or its analogues (Figure 1), such as alisporivir (DEB025), a non-immunosuppressive CsA analogue in phase III clinical trials at Novartis with an EC_50_ value of 0.045 μM (replicon assay), and another non-immunosuppressive CypA inhibitor SCY-635, which is in phase IIa at Scynexis with an EC_50_ value of 0.10 μM (replicon assay) [6,26,27]. However, these analogues might be limited in their applications when considering their synthesis difficulties and poor tolerability [28]. Furthermore, two small molecule anti-HCV CypA inhibitors, F680 and F684, are reported, but the structures of the two compounds have not been made public [28]. Therefore, a novel small molecule anti-HCV agent targeting the host factor of CypA would have extensive prospects and might provide a cure for HCV infection.

In consideration of the important role of CypA in numerous biological processes and related diseases, we are focused on the discovery and application of the CypA inhibitors. In a previous study, we reported a new series of small molecule CypA inhibitors with nanomolar inhibitory potencies which possess a common 1-(benzoyl)-3-(9*H*-fluoren-9-yl)-urea scaffold [29]. In view of the close relationship between CypA and HCV, we wondered if these compounds might possess antiviral activity as set forth. Therefore, a series of experiments reported in this article were performed aiming at discovering and developing a novel kind of anti-HCV agent.

## 2. Results and Discussion

### 2.1. Screening of Compounds for Anti-HCV Activity

Some compounds **1**–**13** which demonstrated potent CypA enzyme inhibitory activity in our previous work were selected for the antiviral tests. Incidentally, four newly synthesized derivatives of these compounds (compounds **14**–**17**) were also incorporated into this assay. As this was a preliminary screen, only one kind of replicon assay was conducted with the results shown in Table 1. In the test, CsA was chosen as the control. The results were encouraging, and among compounds **1**–**17**, compound **1** showed moderately potent anti-HCV activity with an EC_50_ value of 0.92 μM in the replicon assay, which was a little more potent than the control, CsA (EC_50_ = 1.16 μM). Furthermore, it seemed that the fluorene ring was more beneficial for the antiviral activity (compounds **1**, **8** *vs.* **2***‒***4**). More inspiringly, the two newly synthesized compounds **14**, **15** with acetoxyl substituents demonstrated more potent activity than **1** (0.55 and 0.46 μM *vs.* 0.92 μM), which also suggested the chemical modification to be performed as the next step, *viz.* *O*-acylation of the hydroxyl groups on the benzene ring.

**Table 1:** Chemical structures and replicon assay results of compounds **1**–**17** ^a^. 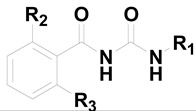

Compound	R_1_	R_2_	R_3_	Anti-HCV JFH1-2a Replicon Assay EC_50_ (μM) ± SD ^b^
**1**	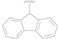	OH	OH	0.92 ± 0.12
**2**	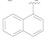	OH	OH	>10
**3**	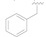	OH	OH	>50
**4**	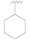	OH	OH	>50
**5**	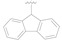	Cl	H	>50
**6**	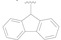	NO_2_	H	>50
**7**	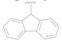	CN	H	>50
**8**	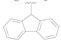	F	H	1.65 ± 0.32
**9**	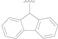	CF_3_	H	>10
**10**	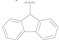	MeS	H	>50
**11**	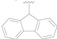	F	F	>50
**12**	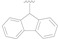	F	MeS	>50
**13**	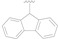	Cl	MeO	>50
**14**	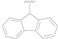	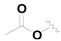	OH	0.55 ± 0.03
**15**	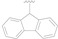	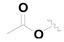	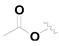	0.46 ± 0.05
**16**	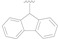	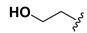	OH	>20
**17**	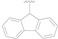	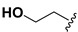	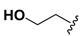	>20
**CsA**				1.16 ± 0.14

^a^ The synthesis and HPLC analysis data of **1**–**13** was according to reference [29]; the synthesis of **16**–**17** was described in the Supporting Information; The experimental method was described in the “Virus Assay” of the “Method”; ^b^ Each value indicates the mean ± SD of three independent experiments.

### 2.2. Spleen Cells Proliferation Inhibition Assay

Considering that CypA regulates numerous biological processes, selectivity should be regard as an important consideration in clinical studies for further drug development, or it may encounter clinical difficulties (e.g., alisporivir, which was put on hold by the FDA due to some side effects). Thus, before the further chemical modification of compound **1** by O-acylation, the immunosuppressive ability was evaluated firstly and CsA was selected as a control in this assay, because an anti-HCV agent via CypA inhibition should possess enough low inhibition activities against proliferation of spleen cells. The result was optimistic, as compound **1** showed quite low inhibition activities (more than 30-fold) against proliferation of spleen cells compared with its anti-HCV activity (Table 2), so it could be regarded as a a non-immunosuppressive lead compound suitable for further research.

**Table 2:** Inhibition of spleen cell proliferation by compound **1** and CsA.

Cmpd	T Spleen Cells IC_50_ (μM) ± SD ^a^	B Spleen Cells IC_50_ (μM) ± SD ^a^
**1**	33.03 ± 5.72	51.93 ± 8.68
**CsA**	0.01 ± 0.005	0.04 ± 0.008

^a^ Each value indicates the mean ± SD of three experiments using one mouse with triplicate sets in each assay. Half-maximal inhibitory concentrations (IC_50_ values) are obtained by log-probit analysis of inhibitory curves using SPSS, a windows statistical software package.

### 2.3. Derivatives Design and Synthesis

**Scheme 1:**
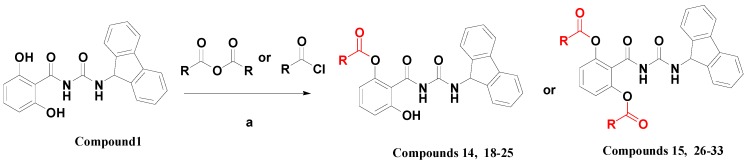
The synthesis of compounds **14**–**15** and **18**–**33**.

In light of the advance of the antiviral activities after derivatization (**14**, **15** *vs.* **1**), we aimed at systematically evaluating the impact of the variation and quantity of acyloxy substituents. Thus varied kinds of substituted acyls (aroyl, straight-chain, branched-chain and cycloalkane substituted acyls) were introduced at the two hydroxyl groups of compound **1**. In totally another 16 compounds **18**–**33** were thus designed and synthesized (Scheme 1), and their chemical structures were shown in Table 3. In general, these esters were prepared by the reaction of compound **1** and the corresponding anhydride or acyl chloride in anhydrous *N*,*N*-dimethylformamide (DMF). The differences between the two reactions are the quantity of the anhydride or acyl chloride used and the reaction temperature. When synthesizing the compounds **18**–**25**, 0.9 equivalents of the anhydride or acyl chloride were used at a temperature below −20 °C, whereas 4 equivalents under room temperature were invariably used for the synthesis of **26**–**33**. The synthesis of compounds **14** and **15** was also performed according to this method. Details of the synthetic procedures and structural characterizations are described in the Experimental Section. All compounds **18**–**33** were confirmed ≥95% purity (Supporting Information, Table S1). 

*Reagents and conditions*: (a) (i) anhydride or acyl chloride, Et_3_N, DMF, r.t. or −20 °C; (ii) r.t.

**Table 3:** Chemical structures of compounds **18**–**33**. 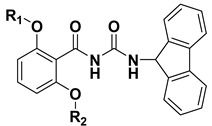

Cmpd	R_1_	R_2_	Cmpd	R_1_	R_2_
**18**	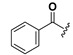	H	**26**	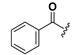	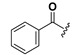
**19**	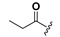	H	**27**	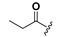	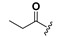
**20**	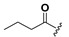	H	**28**	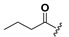	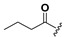
**21**	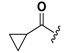	H	**29**	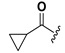	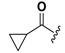
**22**	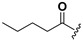	H	**30**	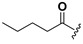	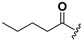
**23**	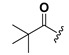	H	**31**	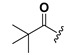	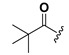
**24**	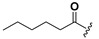	H	**32**	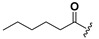	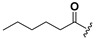
**25**	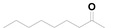	H	**33**	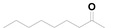	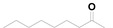

### 2.4. Virus Assays

Virus replicon assays (H77, JFH1 and Con1) and virus assays were performed, with CsA as a control. The results are shown in Table 4. All the ester compounds including compounds **14**, **15** were tested and exhibited more potent antiviral activities than the control in three replicon assays. Furthermore, in the Con-1b replicon assay, most compounds showed similar or more potent activities compared with compound **1**, except **18**, **24**, which were less potent than **1**. The results of the H77-1a and JFH1-2a replicon assays were more exciting, as all the compounds possessed more potent antiviral activities than compound **1**, except **18**, **19**, **24** and **33** in the JFH1-2a assay. Inspiringly, seven compounds (**19**, **20**, **22**, **23**, **25**, **27**, **30**) which were obviously more potent than compound **1** in the virus assays were identified, among which **25** exhibited the most potent anti-HCV effect with an EC_50_ value of 0.19 μM, that was 3.5-fold and 2.1-fold more potent than **1** and CsA, respectively. It should be noted that the antiviral activities of all compounds in the replicon assays were very close to that of the two known small molecular CypA inhibitors F680/F684. Furthermore, all the synthesized compounds showed low cytotoxicity (CC_50_ > 16 μM), indicating that the inhibition of HCV was not due to the death of host cells.

Further studies were conducted using one mutant type JFH1-2a virus strain (S282T, a resistance mutation in the active site that is selected by a series of nucleoside analogues including sofosbuvir, approved by the FDA, that targets HCV NS5B polymerase [30]) to demonstrate the advantage of our compounds **1** and **25** compared with sofosbuvir.

**Table 4:** The anti-HCV activities of compounds **14**, **15**, **18**–**33**.

Cmpd	Anti-HCV Activity				
	EC_50_ (μM) ± SD ^a^				CC_50_ (μM)
	Replicon Assay			Virus Assay	
	H77-1a	JFH1-2a	Con1-1b	JFH1-2a	
**1**	1.11 ± 0.33	0.92 ± 0.12	0.78 ± 0.19	0.67 ± 0.23	>16
**14**	0.77 ± 0.37	0.55 ± 0.03	0.80 ± 0.23	0.71 ± 0.34	>16
**15**	0.80 ± 0.13	0.46 ± 0.05	0.36 ± 0.04	0.74 ± 0.36	>16
**18**	0.60 ± 0.35	1.05 ± 0.01	1.03 ± 0.02	0.70 ± 0.23	>16
**19**	0.38 ± 0.22	0.94 ± 0.07	0.72 ± 0.20	0.47 ± 0.04	>16
**20**	0.98 ± 0.03	0.70 ± 0.02	0.49 ± 0.02	0.28 ± 0.02	>16
**21**	0.57 ± 0.20	0.43 ± 0.08	0.85 ± 0.24	0.78 ± 0.35	>16
**22**	1.00 ± 0.06	0.47 ± 0.02	0.44 ± 0.10	0.44 ± 0.03	>16
**23**	0.67 ± 0.05	0.48 ± 0.02	0.83 ± 0.28	0.53 ± 0.05	>16
**24**	1.05 ± 0.01	1.08 ± 0.05	1.00 ± 0.01	0.74 ± 0.37	>16
**25**	0.85 ± 0.21	0.45 ± 0.08	0.81 ± 0.24	0.19 ± 0.08	>16
**26**	0.53 ± 0.21	0.36 ± 0.03	0.39 ± 0.05	1.09 ± 0.03	>16
**27**	0.52 ± 0.27	0.38 ± 0.03	0.38 ± 0.02	0.42 ± 0.08	>16
**28**	0.79 ± 0.20	0.84 ± 0.32	0.62 ± 0.15	0.68 ± 0.47	>16
**29**	0.52 ± 0.14	0.42 ± 0.14	0.60 ± 0.19	0.74 ± 0.35	>16
**30**	0.73 ± 0.11	0.62 ± 0.05	0.83 ± 0.26	0.47 ± 0.01	>16
**31**	0.87 ± 0.27	0.75 ± 0.29	0.56 ± 0.20	0.84 ± 0.21	>16
**32**	0.63 ± 0.29	0.38 ± 0.05	0.73 ± 0.34	0.74 ± 0.31	>16
**33**	0.54 ± 0.15	0.98 ± 0.01	0.73 ± 0.20	0.95 ± 0.02	>16
**CsA**	1.26 ± 0.20	1.16 ± 0.14	1.27 ± 0.32	0.40 ± 0.11	>8
F680, F684 ^b^			0.20 ^c^		

^a^ Each value indicates the mean ± SD of three independent experiments; ^b^ Small molecular non-immunosuppressive CypA inhibitors currently in preclinical trials at INSERM for HCV therapy [4]; ^c^ Value in the genotype 1b subgenomic replicon assay [26].

As shown in Table 5, the results revealed that both **1** and **25** could maintain their anti-virus activities compared with the results in the assays using wild type JFH1-2a virus strain (0.52 μM *vs.* 0.56 μM and 0.27 μM *vs.* 0.29 μM, respectively), whereas resistance to sofosbuvir was obviously observed (0.22 μM *vs.* 1.14 μM). This indicated that our CypA inhibitors can be regarded as a kind of potential anti-HCV agents.

**Table 5:** The anti-HCV activities of compounds **1** and **25** against mutant type virus strain.

Cmpd	Anti-HCV Activity (Virus Assay)		
		EC_50_ (μM) ± SD ^a^	CC_50_ (μM)
	JFH1-2a (Wild Type)	JFH1-2a (S282T)	
**1**	0.52 ± 0.16	0.56 ± 0.13	>20
**25**	0.27 ± 0.09	0.29 ± 0.07	>20
**Sofosbuvir**	0.22 ± 0.07	1.14 ± 0.21	>20

^a^ Each value indicates the mean ± SD of three independent experiments.

### 2.5. Drug Combination Assay

**Figure 2:**
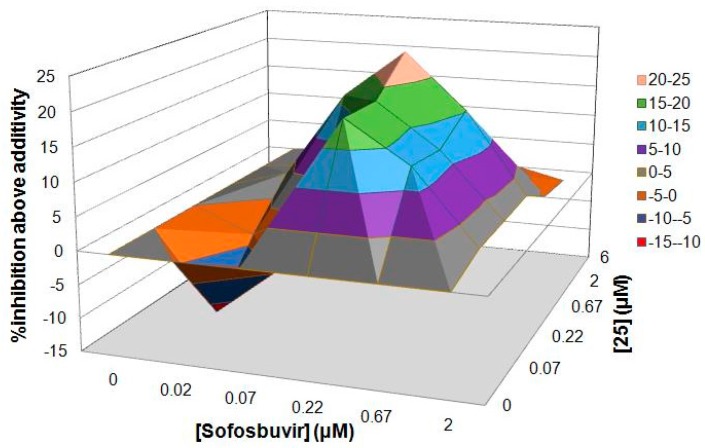
Result of combination study between **25** and sofosbuvir.

Because the inhibition of each step of the virus life cycle can affect the proliferation of a virus, we wondered whether our compounds could be combined with other anti-HCV drugs currently used in hepatitis C treatment. To investigate this hypothesis, our most potent compound **25** and sofosbuvir were employed in this study. The assay of the combination was evaluated with a HCV cell culture (JFH1-2a) system with a luciferase reporter gene. To determine whether the effects of the combination were synergistic, additive, or antagonistic, the data were analyzed using the method of Prichard and Shipman [31]. The results revealed that the **25**‒sofosbuvir combination had an ntiviral effect that was more potent than the theoretical additive effects, as the maximal percent synergy extended to the range of 20% to 25%, which supported the notion that this combination was indeed synergistic (Figure 2). Nearly no evidence of significant antiviral antagonism was observed with the tested doses, as the maximal percent antagonism did not exceed almost 10%.

### 2.6. CypA Enzyme Inhibition Assay

The standard chymotrypsin-coupled assay results (Table 6) indicated that all the compounds remarkably reduced CypA inhibitory activities *in vitro* after the acylation of the hydroxyl groups. Compared with **1** (CypA enzyme inhibition: IC_50_ = 0.032 ± 0.002 μM), 89% of the *O*-acylated esters (**16**/**18)** showed found no CypA inhibition activity (IC_50_ > 5 μM), and only the monoacetylated compound **14** and monobenzoylated compound **18** maintained low CypA inhibitory activities (IC_50_ = 2.43 ± 0.50 and 1.99 ± 0.47 μM, respectively). In consideration of the potent anti-HCV activity and the ester structure features, we speculated that this kind of ester was not the active form in the antiviral process and there was probably a prodrug mechanism in these series of compounds. Therefore, several tests were conducted in the next step to make a preliminary verification of this hypothesis.

**Table 6:** Enzyme inhibition activities of compounds **14**, **15**, **18**–**33**.

Cmpd	Enzyme Inhibition Assay IC_50_ (μM) ± SD ^a^
**1**	0.032 ± 0.002
**14**	2.43 ± 0.50
**18**	1.99 ± 0.47
**15** and **19–33**	>5

^a^ Each value indicates the mean ± SD of five independent experiments.

### 2.7. Preliminary Verification of the Hypothetical Prodrug Mechanism

**Figure 3:**
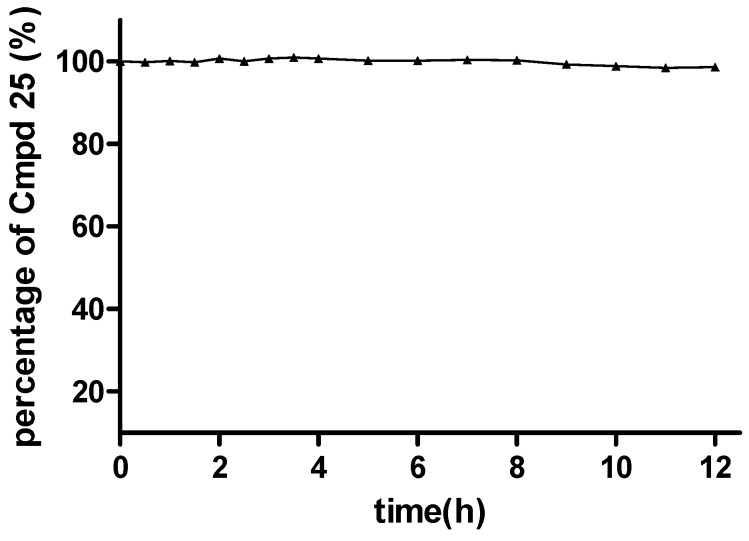
Stability of **25** under pH 7.4 conditions.

In this section, the most potent compound **25** in the virus assay was chosen for further study to make a preliminary verification of the hypothetical prodrug mechanism. Specifically, a solution stability assay and a simple pharmacokinetic test were performed, using the experimental methods described in the Supporting Information. The solution stability assay results showed that the ester moiety of **25** was quite stable in PBS buffer at pH 7.4. For example, after 12 h in the PBS buffer, 98.6% of **25** still remained in the original form (Figure 3 and Supporting Information, Table S2), indicating that this series of compounds possessed sufficient stability under *in vitro* physiological conditions.

Moreover, the pharmacokinetic test result indicated that **25** exhibited a high release rate of **1** *in vivo*. After the intravenous injection of **25** (2 mg∙kg^−1^) into rats, both **25** and **1** could be immediately detected in the plasma. However, the plasma concentration of **1** was much higher than that of **25**. After 5 min, the plasma concentration of **1** was 904.5 ± 291.9 ng∙mL^−1^ (mean ± SD), whereas the plasma concentration of **25** was only 20.47 ± 9.99 ng∙mL^−1^ (mean ± SD), indicating that **25** could be hydrolyzed into **1** rapidly (Supporting Information, Table S3). Furthermore, the AUC**_1_**/AUC**_25_** value was about 60, which also demonstrated that the main form of **25** existing in body circulation was compound **1**.

## 3. Experimental Section

### 3.1. General Information

The reagents were purchased from Adamas-beta (Shanghai, China), J & K (Beijing, China), Energy Chemical (Shanghai, China) and TCI (Shanghai, China) and used without further purification. Analytical thin-layer chromatography (TLC) was performed on HSGF 254 plates (150–200 µm thickness, Yantai Huiyou Company, Yantai, China). Yields were not optimized. Melting points were measured in capillary tubes on a SGW X-4 melting point apparatus (Shanghai Jingmiyiqi Company, Shanghai, China) without correction. Nuclear magnetic resonance (NMR) spectra were recorded on a AVANCE 400 NMR (Bruker, Karlsruhe, Germany). Chemical shifts are reported in parts per million (ppm, δ) downfield from tetramethylsilane (TMS) used as the internal standard. Proton coupling patterns are described as singlet (s), doublet (d), triplet (t), quartet (q), multiplet (m), and broad (br). Low-and high-resolution mass spectra (LRMS and HRMS) were obtained with electric ionization (EI) and electrospray ionization (ESI) on Finnigan MAT-95 (Bremen, Germany) and LCQ-DECA (Bremen, Germany) spectrometers.

1-(2-Acetoxy-6-hydroxybenzoyl)-3-(9H-fluoren-9-yl)-urea (**14**). To a solution of **1** (0.9 g) and triethylamine (0.35 mL) in anhydrous DMF (25 mL), was added acetic anhydride (0.21 mL) with stirring at −20 °C. The mixture was stirred at room temperature for 12 h, and then heated to 40 °C with stirring for another 24 h. The reaction mixture was poured into H_2_O (500 mL), extracted with dichloromethane (DCM, 200 mL) three times. The organic layers were combined, dried with anhydrous sodium sulfate, and concentrated to dryness. The residue was purified by flash column chromatography on silica gel, eluted with a mixture of THF/DCM/petroleum ether (PE) (1:5:3, *v*/*v*/*v*), to afford **14** (161 mg, 16%) as a white solid: mp 215–225 °C; ^1^H-NMR (DMSO-*d*_6_) δ: 10.84 (s, 1H), 10.45 (s, 1H), 8.69 (s, 1H), 7.89 (d, *J* = 7.5 Hz, 2H), 7.62 (t, *J* = 7.5 Hz, 2H), 7.46 (t, *J* = 7.4 Hz, 2H), 7.37 (t, *J* = 7.4 Hz, 2H), 7.27 (t, *J* = 8.2 Hz, 1H), 6.80 (d, *J* = 8.3 Hz, 1H), 6.63 (d, *J* = 8.0 Hz, 1H), 5.97 (d, *J* = 7.9 Hz, 1H), 2.13 (s, 3H); ESI-MS *m*/*z* 401.0 [M − H]^−^; HRMS (ESI) *m*/*z* calcd C_23_H_18_N_2_O_5_ [M − H]^−^ 401.1137, found 401.1143.

*1-(2-Benzoyloxy-6-hydroxybenzoyl)-3-(9H-fluoren-9-yl)-urea* (**18**). Prepared in the same manner as described for **14**, but the acetic anhydride was replaced by benzoyl chloride: mp 211–214 °C; ^1^H-NMR (DMSO-*d*_6_) δ: 11.07 (s, 1H), 10.45 (s, 1H), 8.61 (s, 1H), 8.01 (d, *J* = 7.4 Hz, 2H), 7.85 (t, *J* = 7.7 Hz, 2H), 7.78 (t, *J* = 7.4 Hz, 1H), 7.58 (t, *J* = 7.7 Hz, 2H), 7.40 (dd, *J* = 14.5, 7.0 Hz, 2H), 7.38–7.30 (m, 3H), 7.26 (t, *J* = 7.4 Hz, 2H), 6.84 (t, *J* = 8.9 Hz, 2H), 5.91 (d, *J* = 8.2 Hz, 1H); ESI-MS *m*/*z* 463.0 [M − H]^−^; HRMS (ESI) *m/z* calcd C_28_H_20_N_2_O_5_ [M − H]^−^ 463.1294, found 463.1299.

*1-(2-Hydroxy-6-propionyloxybenzoyl)-3-(9H-fluoren-9-yl)-urea* (**19**). Prepared in the same manner as described for **14**, but the acetic anhydride was replaced by propionic anhydride: mp 206–216 °C; ^1^H-NMR (acetone-*d*_6_) δ: 9.72 (s, 1H), 8.71 (d, *J* = 8.1 Hz, 1H), 7.86 (d, *J* = 7.5 Hz, 2H), 7.70 (d, *J* = 7.4 Hz, 2H), 7.46 (t, *J* = 7.4 Hz, 2H), 7.43–7.34 (m, 3H), 6.89 (d, *J* = 8.4 Hz, 1H), 6.75 (d, *J* = 8.1 Hz, 1H), 6.11 (d, *J* = 8.1 Hz, 1H), 2.60 (q, *J* = 7.5 Hz, 2H), 1.23–1.12 (m, 3H); ESI-MS *m*/*z* 415.0 [M − H]^−^; HRMS (ESI) *m/z* calcd C_24_H_20_N_2_O_5_ [M − H]^−^ 415.1294, found 415.1299.

*1-(2-Hydroxy-6-n-butyryloxybenzoyl)-3-(9H-fluoren-9-yl)-urea* (**20**). Prepared in the same manner as described for **14**, but the acetic anhydride was replaced by butyric anhydride: mp 204–210 °C; ^1^H-NMR (acetone-*d*_6_) δ: 8.71 (s, 1H), 7.87 (d, *J* = 7.5 Hz, 2H), 7.71 (d, *J* = 7.5 Hz, 2H), 7.47 (t, *J* = 7.4 Hz, 2H), 7.44–7.36 (m, 3H), 6.91 (d, *J* = 7.7 Hz, 1H), 6.75 (d, *J* = 7.9 Hz, 1H), 6.12 (d, *J* = 8.2 Hz, 1H), 2.56 (t, *J* = 7.3 Hz, 2H), 1.76–1.64 (m, 2H), 0.96 (t, *J* = 7.4 Hz, 3H); ESI-MS *m*/*z* 429.1 [M − H]^−^; HRMS (ESI) *m/z* calcd C_25_H_22_N_2_O_5_ [M − H]^−^ 429.1450, found 429.1456.

*1-(2-Cyclopropyl formyloxy-6-hydroxybenzoyl)-3-(9H-fluoren-9-yl)-urea* (**21**). Prepared in the same manner as described for **14**, but the acetic anhydride was replaced by cyclopropanecarboxylic acid chloride: mp 194–199 °C; ^1^H-NMR (DMSO-*d*_6_) δ: 10.85 (s, 1H), 10.36 (s, 1H), 8.71 (s, 1H), 7.90 (d, *J* = 7.6 Hz, 2H), 7.61 (d, *J* = 7.5 Hz, 2H), 7.47 (t, *J* = 7.4 Hz, 2H), 7.38 (t, *J* = 7.5 Hz, 2H), 7.25 (t, *J* = 8.2 Hz, 1H), 6.79 (d, *J* = 8.2 Hz, 1H), 6.63 (d, *J* = 8.2 Hz, 1H), 6.00 (d, *J* = 8.1 Hz, 1H), 1.75 (d, *J* = 4.8 Hz, 1H), 0.99–0.83 (m, 4H); EI-MS *m/z* 428.1(M^+^); 165.1 (100%); HRMS (EI) *m/z* calcd C_25_H_20_ N_2_O_5_ (M^+^) 428.1372, found 428.1373.

*1-(2-Hydroxy-6-valeryloxybenzoyl)-3-(9H-fluoren-9-yl)-urea* (**22**). Prepared in the same manner as described for **14**, but acetic anhydride was replaced by valeric anhydride: mp 193–199 °C; ^1^H-NMR (acetone-*d*_6_) δ: 8.73 (d, *J* = 6.2 Hz, 1H), 7.86 (d, *J* = 7.6 Hz, 2H), 7.70 (d, *J* = 7.5 Hz, 2H), 7.46 (t, *J* = 7.6 Hz, 2H), 7.43–7.35 (m, 3H), 6.89 (d, *J* = 8.3 Hz, 1H), 6.73 (d, *J* = 8.1 Hz, 1H), 6.11 (d, *J* = 8.1 Hz, 1H), 2.57 (t, *J* = 7.4 Hz, 2H), 1.64 (dt, *J* = 15.2, 7.5 Hz, 2H), 1.37 (dq, *J* = 14.7, 7.4 Hz, 2H), 0.86 (t, *J* = 7.4 Hz, 3H); ESI-MS *m*/*z* 443.0 [M − H]^−^; HRMS (ESI) *m/z* calcd C_26_H_24_N_2_O_5_ [M − H]^−^ 443.1607, found 443.1612.

*1-(2-Hydroxy-6-trimethylacetoxyloxybenzoyl)-3-(9H-fluoren-9-yl)-urea* (**23**). Prepared in the same manner as described for **14**, but the acetic anhydride was replaced by trimethylacetic anhydride: mp 187–189 °C; ^1^H-NMR (acetone-*d*_6_) δ: 9.71 (s, 1H), 8.76 (s, 1H), 7.86 (d, *J* = 7.5 Hz, 2H), 7.68 (d, *J* = 7.4 Hz, 2H), 7.46 (t, *J* = 7.4 Hz, 2H), 7.37 (dd, *J* = 13.2, 7.3 Hz, 3H), 6.87 (d, *J* = 8.2 Hz, 1H), 6.69 (d, *J* = 8.1 Hz, 1H), 6.11 (d, *J* = 7.9 Hz, 1H), 1.31 (s, 9H); ESI-MS *m*/*z* 443.0 [M − H]^−^; HRMS (ESI) *m/z* calcd C_26_H_24_N_2_O_5_ [M − H]^−^ 443.1607, found 443.1612.

*1-(2-Hexanoyloxy-6-hydroxybenzoyl)-3-(9H-fluoren-9-yl)-urea* (**24**). Prepared in the same manner as described for **14**, but the acetic anhydride was replaced by hexanoic anhydride: mp 200–222 °C; ^1^H-NMR (DMSO-*d*_6_) δ*:* 11.09 (s, 1H), 10.42 (s, 1H), 8.73 (s, 1H), 7.89 (d, *J* = 7.5 Hz, 2H), 7.60 (d, *J* = 7.4 Hz, 2H), 7.46 (t, *J* = 7.3 Hz, 2H), 7.36 (t, *J* = 7.3 Hz, 2H), 7.25 (t, *J* = 8.2 Hz, 1H), 6.77 (d, *J* = 8.3 Hz, 1H), 6.57 (d, *J* = 8.0 Hz, 1H), 5.98 (d, *J* = 7.8 Hz, 1H), 2.39 (t, *J* = 7.4 Hz, 2H), 1.48 (dd, *J* = 14.6, 7.3 Hz, 2H), 1.22–1.03 (m, 4H), 0.72 (t, *J* = 7.0 Hz, 3H). ; ESI-MS *m*/*z* 457.0 [M − H]^−^; HRMS (ESI) *m/z* calcd C_27_H_26_N_2_O_5_ [M − H]^−^ 457.1763, found 457.1769.

*1-(2-Hydroxy-6-n-Octanoyloxybenzoyl)-3-(9H-fluoren-9-yl)-urea* (**25**). Prepared in the same manner as described for **14**, but the acetic anhydride was replaced by *n*-octanoic anhydride: mp 184–189 °C; ^1^H-NMR (CDCl_3_) δ: 11.56 (s, 1H), 9.45 (s, 1H), 8.48 (d, *J* = 8.4 Hz, 1H), 7.73 (d, *J* = 7.6 Hz, 2H), 7.65 (d, *J* = 7.5 Hz, 2H), 7.43 (dd, *J* = 12.1, 4.8 Hz, 3H), 7.34 (t, *J* = 7.5 Hz, 2H), 6.89 (d, *J* = 8.4 Hz, 1H), 6.69 (d, *J* = 8.1 Hz, 1H), 6.16 (d, *J* = 8.4 Hz, 1H), 2.81 (t, *J* = 7.4 Hz, 2H), 1.85 (dt, *J* = 15.1, 7.4 Hz, 2H), 1.53–1.28 (m, 8H), 0.91 (t, *J* = 6.8 Hz, 3H); EI-MS *m/z* 486.2 (M^+^); 165.1 (100%); HRMS (EI) *m/z* calcd C_29_H_30_N_2_O_5_ (M^+^) 486.2155, found 486.2157.

*1*-[2,6-Bis-(acetoxy)benzoyl]-*3-(9H-fluoren-9-yl)-urea* (**15**). To a solution of **1** (0.9 g) and triethylamine (1.38 mL) in anhydrous DMF (15 mL), was added acetic anhydride (0.93 mL) with stirring at room temperature for 12 h. The reaction mixture was poured into H_2_O (300 mL), and extracted with DCM (120 mL) three times. The organic layers were combined, dried with anhydrous sodium sulfate, then concentrated to dryness. The residue was purified by flash column chromatography on silica gel, eluted with a mixture of THF/DCM/PE (1:5:3, *v*/*v*/*v*), to afford **15** (760 mg, 69%) as a white solid: mp 191–194 °C; ^1^H-NMR (acetone-*d*_6_) δ: 8.64 (s, 1H), 7.86 (d, *J* = 7.5 Hz, 2H), 7.69 (d, *J* = 7.5 Hz, 2H), 7.56 (t, *J* = 8.3 Hz, 1H), 7.46 (t, *J* = 7.4 Hz, 2H), 7.38 (t, *J* = 7.2 Hz, 2H), 7.18 (t, *J* = 8.7 Hz, 2H), 6.10 (d, *J* = 8.1 Hz, 1H), 2.24 (s, 6H); ESI-MS *m*/*z* 445.0 [M + H]^+^; HRMS (ESI) *m/z* calcd C_25_H_20_N_2_O_6_ [M + Na]^+^ 467.1219, found 467.1214.

1-[2,6-Bis-(benzoyloxy)benzoyl]-*3-(9H-fluoren-9-yl)-urea* (**26**). Prepared in the same manner as described for **15**, but the acetic anhydride was replaced by benzoyl chloride: mp 211–213 °C; ^1^H-NMR (acetone-*d*_6_) δ: 10.33 (s, 1H), 8.54 (d, *J* = 7.0 Hz, 1H), 8.18 (d, *J* = 8.2 Hz, 4H), 7.84–7.68 (m, 5H), 7.62 (t, *J* = 7.7 Hz, 4H), 7.48 (d, *J* = 8.3 Hz, 2H), 7.37 (t, *J* = 7.5 Hz, 2H), 7.31 (d, *J* = 7.4 Hz, 2H), 7.21 (t, *J* = 7.4 Hz, 2H), 5.96 (d, *J* = 8.3 Hz, 1H); EI-MS *m/z* 568.2(M^+^); 105.0 (100%); HRMS (ESI) *m/z* calcd C_35_H_24_N_2_O_6_ [M + Na]^+^ 591.1532, found 591.1523.

*1*-[2,6-Bis-(propionyloxy)benzoyl]-3-(9H*-fluoren-9-yl)-urea* (**27**). Prepared in the same manner as described for **15**, but the acetic anhydride was replaced by propionic anhydride: mp 165–167 °C; ^1^H-NMR (acetone-*d*_6_) δ: 9.89 (s, 1H), 8.67 (s, 1H), 7.87 (d, *J* = 7.6 Hz, 2H), 7.68 (d, *J* = 7.5 Hz, 2H), 7.58 (t, *J* = 8.3 Hz, 1H), 7.48 (t, *J* = 7.3 Hz, 2H), 7.43–7.35 (m, 2H), 7.20 (d, *J* = 8.3 Hz, 2H), 6.11 (d, *J* = 8.1 Hz, 1H), 2.59 (q, *J* = 7.5 Hz, 4H), 1.18 (t, *J* = 7.5 Hz, 6H); EI-MS *m/z* 472.2(M^+^); 165.1 (100%); HRMS (ESI) *m/z* calcd C_27_H_24_N_2_O_6_ [M + Na]^+^ 495.1532, found 495.1527.

1-[2,6-Bis-(n-butyryloxy)benzoyl]-3-(9H-fluoren-9-yl*)-urea* (**28**). Prepared in the same manner as described for **15**, but the acetic anhydride was replaced by butyric anhydride: mp 186–188 °C; ^1^H-NMR (a*d*_6_) δ: 9.90 (s, 1H), 8.68 (s, 1H), 7.86 (d, *J* = 7.5 Hz, 2H), 7.67 (d, *J* = 7.5 Hz, 2H), 7.56 (t, *J* = 8.3 Hz, 1H), 7.47 (t, *J* = 7.3 Hz, 2H), 7.38 (td, *J* = 7.5, 1.0 Hz, 2H), 7.18 (d, *J* = 8.3 Hz, 2H), 6.10 (d, *J* = 8.1 Hz, 1H), 2.53 (t, *J* = 7.3 Hz, 4H), 1.78–1.62 (m, 4H), 0.96 (t, *J* = 7.4 Hz, 6H); EI-MS *m/z* 500.2 (M^+^); 165.1 (100%); HRMS (ESI) *m/z* calcd C_29_H_28_N_2_O_6_ [M + Na]^+^ 523.1845, found 523.1840.

1-[2,6-Bis-(cyclopropylformyloxy)benzoyl]-3-(9H-fluoren*-9-yl)-urea* (**29**). Prepared in the same manner as described for **15**, but the acetic anhydride was replaced by cyclopropanecarboxylic acid chloride: mp 183–186 °C; ^1^H-NMR (CDCl_3_) *δ*: 8.50 (d, *J* = 8.0 Hz, 1H), 7.92 (s, 1H), 7.73 (d, *J* = 7.5 Hz, 2H), 7.65 (d, *J* = 7.5 Hz, 2H), 7.49 (t, *J* = 8.2 Hz, 1H), 7.43 (t, *J* = 7.4 Hz, 2H), 7.33 (t, *J* = 7.5 Hz, 2H), 7.08 (d, *J* = 8.2 Hz, 2H), 6.17 (d, *J* = 8.7 Hz, 1H), 1.84 (td, *J* = 7.8, 4.1 Hz, 2H), 1.21–1.13 (m, 4H), 1.08–0.99 (m, 4H); EI-MS *m/z* 496.2(M^+^); 165.1 (100%); HRMS (ESI) *m/z* calcd C_29_H_24_N_2_O_6_ [M + Na]^+^519.1532, found 519.1523.

1-[2,6-Bis-(valeryloxy)benzoyl]-3-(9H-fluoren-9-yl)-urea (**30**). Prepared in the same manner as described for **15**, but the acetic anhydride was replaced by valeric anhydride: mp 172–174 °C; ^1^H-NMR (CDCl_3_) δ: 8.47 (d, *J* = 8.3 Hz, 1H), 8.01 (s, 1H), 7.72 (d, *J* = 7.5 Hz, 2H), 7.62 (d, *J* = 7.5 Hz, 2H), 7.49 (t, *J* = 8.2 Hz, 1H), 7.42 (t, *J* = 7.5 Hz, 2H), 7.31 (t, *J* = 7.4 Hz, 2H), 7.07 (d, *J* = 8.2 Hz, 2H), 6.13 (d, *J* = 8.4 Hz, 1H), 2.55 (t, *J* = 7.5 Hz, 4H), 1.75–1.63 (m, 4H), 1.38 (tt, *J* = 11.3, 5.8 Hz, 4H), 0.91 (t, *J* = 7.3 Hz, 6H); EI-MS *m/z* 528.2(M^+^); 165.1 (100%); HRMS (ESI) *m/z* calcd C_31_H_32_N_2_O_6_ [M + Na]^+^ 551.2158, found 551.2153.

1-[2,6-Bis-(trimethylacetoxyloxy)benzoyl]-*3-(9H-fluoren-9-yl)-urea* (**31**). Prepared in the same manner as described for **15**, but the acetic anhydride was replaced by trimethylacetic anhydride: mp 202–205 °C; ^1^H-NMR (CDCl_3_) δ: 8.55 (d, *J* = 8.9 Hz, 1H), 7.71 (d, *J* = 7.5 Hz, 2H), 7.50 (t, *J* = 7.6 Hz, 2H), 7.49–7.37 (m, 3H), 7.28 (dd, *J* = 14.4, 6.9 Hz, 2H), 7.04 (d, *J* = 8.3 Hz, 2H), 6.13 (d, *J* = 8.7 Hz, 1H), 1.32 (s, 18H); EI-MS *m/z* 528.2(M^+^); 165.1 (100%); HRMS (ESI) *m/z* calcd C_31_H_32_N_2_O_6_ [M + Na]^+^ 551.2158, found 551.2153.

1-[2,6-Bis-(hexanoyloxy)benzoyl]-*3-(9H-fluoren-9-yl)-urea* (**32**). Prepared in the same manner as described for **15**, but the acetic anhydride was replaced by hexanoic anhydride: mp 162–165 °C; ^1^H-NMR (CDCl_3_) δ: 8.46 (d, *J* = 8.0 Hz, 1H), 7.93 (s, 1H), 7.72 (d, *J* = 7.6 Hz, 2H), 7.62 (d, *J* = 7.4 Hz, 2H), 7.50 (t, *J* = 8.2 Hz, 1H), 7.42 (t, *J* = 7.4 Hz, 2H), 7.31 (dd, *J* = 14.1, 6.7 Hz, 2H), 7.07 (d, *J* = 8.3 Hz, 2H), 6.14 (d, *J* = 8.2 Hz, 1H), 2.54 (t, *J* = 7.6 Hz, 4H), 1.69 (dd, *J* = 14.6, 7.2 Hz, 4H), 1.30 (dd, *J* = 20.4, 5.5 Hz, 8H), 0.88 (t, *J* = 6.8 Hz, 6H); EI-MS *m/z* 556.3(M^+^); 165.1 (100%); HRMS (ESI) *m/z* calcd C_33_H_36_N_2_O_6_ [M + Na]^+^ 579.2471, found 579.2466.

1-[2,6-Bis-(n-Octanoyloxy)benzoyl]-*3-(9H-fluoren-9-yl)-urea* (**33**). Prepared in the same manner as described for **15**, but the acetic anhydride was replaced by *n*-octanoic anhydride: mp 152–153 °C; ^1^H-NMR (CDCl_3_) δ: 8.48 (d, *J* = 8.3 Hz, 1H), 8.17 (s, 1H), 7.71 (t, *J* = 8.3 Hz, 2H), 7.61 (d, *J* = 7.5 Hz, 2H), 7.51–7.45 (m, 1H), 7.42 (t, *J* = 7.5 Hz, 2H), 7.31 (t, *J* = 7.5 Hz, 2H), 7.07 (d, *J* = 8.3 Hz, 2H), 6.12 (d, *J* = 8.4 Hz, 1H), 2.54 (t, *J* = 7.6 Hz, 4H), 1.77–1.63 (m, 4H), 1.34–1.17 (m, 16H), 0.87 (t, *J* = 6.3 Hz, 6H); HRMS (ESI) *m/z* calcd C_37_H_44_N_2_O_6_ [M + H]^+^ 613.3278, found 613.3267.

### 3.2. Biological Assays

#### 3.2.1. Spleen Cells Proliferation Inhibition Assay

Preparation of Splenocyte Suspension: The spleen was aseptically taken from female ICR mice strains (6–8 weeks-old, 20 ± 2 g), crushed gently and separated into single cells by squeezing in 20 mL RPMI 1640 medium (Invitrogen, shanghai, China). The cells obtained were passed through 70 μM nylon cell strainer and centrifuged at 1000 rpm∙min^−1^ for 5 min at RT. Pellets were added into 10 mL erythrocytes lysis buffer (150 mM NH_4_Cl, 10 mM KHCO_3_ and 0.1 mM EDTA) followed by centrifugation to remove erythrocytes. After washing twice with complete medium (RPMI 1640 containing 100 U∙mL^−1^ of penicillin, 100 U∙mL^−1^ of streptomycin and 10% FBS), they were re-suspended in the complete medium and used for cell culture.

Proliferation Assay of Spleen Cells: During this assay, CsA was used as a control for evaluation of compound **1** inhibition activity. 5 × 10^5^ spleen cells were cultured in 96-well flat-bottom microplates (Corning Incorporatrd Coring, New York, NY, USA) in RPMI 1640 complete medium containing 5 μg∙mL^−1^ of concanavalin A (Con A) in the presence of increasing concentration of compounds: CsA (0.01, 0.1, 0.5, 1, 2, 5, 8, 10, 100 μM); compound **1** (0.01, 0.1, 0.5, 1, 2, 5, 8, 10, 100 μM). After 72 h of incubation, the cell growth was evaluated with MTT assay. The assay plates were added 10 μL of 5 mg∙mL^−1^ MTT per well and incubated for 4 h. The formazan generated was solubilized with 100 μL of solution (10% SDS, 5% isobutyl alcohol, and 10 mM HCl) after incubated at 37 °C overnight. The absorption at 580 nm relative to 680 nm was measured by SpectraMax Plus 384 Reader (Molecular Devices, Shanghai, China).

#### 3.2.2. Virus Assay

Viral Inhibition Assay: The HCV virus assay was constructed by using the method according to [32,33] with a little modification. The methods of establishing the HCV virus and subgenomic replicon cell lines were described in the Supporting Information. Huh 7.5.1 cells were seeded in 96-well plates at a density of 2 × 10^4^ cells per well at 37 °C overnight. All of the synthetic compounds were diluted with dimethyl sulfoxide (DMSO) to 10 mM of stock solution. The initial concentration of compounds was 20 μM and then diluted at a gradient of 1:4 to the concentrations ranging from 20 μM to 0.02 μM containing 0.5% DMSO. For the HCV virus assay, serial diluted compounds were mixed with a certain titer of JFH-1 virus, and the final concentration of JFH-1 virus titer was diluted to the numbers of relative luminescence units (RLU) ranging from 20,000 to 50,000 RLU and then added to the Huh 7.5.1 cells. For the HCV replicon system, serially diluted compounds was added to the Huh 7.5.1 replicon cells and the cells were cultured for 2 days at 37 °C. Then the cells were harvested and the luminescence was detected following the manufacturer’s protocol for the Renilla-Glo™ Luciferase Assay System. EC_50_ was the concentration of compound at which the HCV luminescence level in the Huh7.5.1 cells is reduced by 50%. Each data point represents the average of two replicates in cell culture. The values of EC_50_ were plotted by the GraphPad Prism 5 software.

Cell Proliferation Assay: Huh 7.5.1 cells were seeded at a density of 2 × 10^4^ cells per well in 96-well plates overnight. The cells were respectively incubated with serial diluted compounds for 48 h, and the viability of Huh7.5.1 cells was determined in 96-well tissue culture plates using cell proliferation reagent WST-1 (Roche, Basel, Switzerland). The absorbance (OD450 /reference OD630) was measured to detect the cytotoxicity of compounds, according to the manufacturer’s protocol of Cell Proliferation Reagent WST-1.

#### 3.2.3. Drug Combination Assay

Huh 7.5.1 cells were seeded at 1 × 10^4^ per well in 96-well microtiter plates. After overnight culture, Huh 7.5.1 cells were infected with JFH-1 virus with luciferase reporter gene and treated with various concentrations of **25** alone, Sofosbuvir alone, or in combinations for 24 h. Antiviral activities were determined by measuring the reduction of luciferase activities in the cells. 3D-analysis of combinations on JFH1 infection was evaluated using the method of Prichard and Shipman [31]. Combination studies for each pair of compounds were performed in triplicate. The theoretical additive surface is subtracted from the actual experimental surface, resulting in a horizontal surface that equals the zero plane when the combination is additive. A surface raising more than 25% above the zero plane indicates a synergistic effect of the combination and a surface dropping lower than 25% below the zero plane indicates antagonism.

#### 3.2.4. CypA Enzyme Inhibition Assay

The PPIase activity of CypA was conducted in a coupled assay with chymotrypsin using the synthetic tetrapeptide *N*-succinyl-Ala-Ala-Pro-Phe-*p*-nitroanilide (Sigma, Shanghai, China) as described in [34] with small modifications. The assay buffer (20 mM Tris-HCl, 50 mM NaCl, pH 7.8) and CypA were precooled at 4 °C and mixed with 0.4 mg∙mL^−1^ chymotrypsin (20 mg∙mL^−1^ in 1 mM HCl). The reaction was initiated by adding peptide substrate (2 mg∙mL^−1^ in 500 mM LiCl in tetrahydrofuran (THF)). After a delay from the onset of mixing (usually 6 s), the *cis*-to-*trans* isomerization of the Ala-Pro peptide bond, coupled with the chymotryptic cleavage of the *trans* peptide, then the *p*-nitroanilide was quantified by the increase in absorbance at 380 nm for 60 s by a U-3010 spectrophotometer. Absorbance readings were collected every 0.1 s and the temperature was controlled at 4 °C during the assay. The progress curves were analyzed by nonlinear least-squares fit. The inhibition assays of compounds were performed in the same manner as above-mentioned. 10 µM of test compounds in DMSO, at various concentrations, was added to the CypA solution in the assay buffer. After being preincubated for 1 h at 4 °C, the assay was started by the addition of chymotrypsin and the substrate. To calculate the half-maximal inhibitory concentration (IC_50_), a percent of the remaining PPIase activity was plotted against the common logarithm of the compound concentration, and the data were fitted using the sigmoidal fitting model of Origin 7.0 software.

## 4. Conclusions

Considering the important role of CypA for HCV proliferation, we were enlightened and prompted to screen a series of compounds previously reported as CypA inhibitors and their derivatives. Fortunately, the small molecule CypA inhibitor **1** and its O-acetylated derivatives **14**, **15** with potent anti-HCV activity were discovered. The subsequent assessment showed that compound **1** can be regarded as a non-immunosuppressive lead compound for further research, hence a series of acylated derivatives of the hydroxyl groups of compound **1** were prepared to give another 16 derivatives. Among these compounds, **25** exhibited the most potent antiviral activities in replicon and virus assays but low cytotoxicity (CC_50_ > 16 μM) and hERG (the human *Ether-à-go-go*-related gene) cardiac toxicity (hERG: IC_50_ > 15 μM, the experimental method was described in the Supporting Information). Furthermore, the solution stability assay and the simple pharmacokinetic test together with the CypA inhibition result preliminarily indicated that compound **25** was probably a prodrug of **1**. The proof was set forth as follows: (1) **25** was inactive at the enzyme level (CypA enzyme inhibition: IC_50_ > 5 μM), whereas it was active at the cellular level (virus assay: EC_50_ = 0.19 μM); (2) **25** showed sufficient stability *in vitro* (stable in PBS buffer at pH 7.4 after 12 h), but could rapidly release **1** *in vivo* (AUC**_1_**/AUC**_A8_** ≈ 60). However, further study is still necessary to explicitly demonstrate the hypothesized prodrug mechanism. To the best of our knowledge, compound **25** is probably the most potent small molecule anti-HCV agent in virus assay via CypA inhibitory mechanism. In conclusion, our present study indicated that compounds **1** and **25** could be used as a new class of potent anti-HCV agents and the further pre-clinical studies of the two compounds are currently underway in our laboratory.

## Supplementary Materials

Supplementary materials can be accessed at: http://www.mdpi.com/1420-3049/20/06/10342/s1.
