# Supplementary material for: Discovery of Novel Small Molecule Anti-HCV Agents via the CypA Inhibitory Mechanism Using O-Acylation-Directed Lead Optimization
Source: Molecules. 2015 Jun 4;20(6):10342–59. doi: 10.3390/molecules200610342 (PMC6272715; doi:10.3390/molecules200610342)
Supplement: Supplementary file 1 [file molecules-20-10342-s001.pdf]

Supplementary Material

Synthesis of Compounds **16**,**17** S1
HPLC Analysis Data of Compounds **14**–**33** S4
Establishment of HCV Virus and Subgenomic Replicon Cell Lines S4
Solution Stability Assay S5
Pharmacokinetic Studies S6
hERG Cardiac Toxicity Assay of Compound **25** S6
Supporting References S8

Synthesis of Compounds 16, 17

Scheme 1S depicts the sequence of reactions that led to the preparation of compounds **16**, **17** using 2,6-difluorobenzonitrile (**35**) as the starting material. 2,6-difluorobenzonitrile (**35**) was reacted by refluxing with sodium 2-benzyloxy-ethanolate in DMSO, which afforded 2-(2-benzyl-oxyethoxy)-6-fluorobenzonitrile (**36**) or 2,6-bis-(2-benzyloxy-ethoxy)-benzonitrile (**41**). To synthesize compound **16**, **36** was reacted by refluxing with sodium benzyloxide in DMSO to afford 2-benzyloxy-6-(2-benzyloxyethoxy)-benzonitrile (**37**), which was subsequently hydrolyzed to give 2-benzyloxy-6-(2-benzyloxyethoxy)-benzamide (**38**) [1]. The key intermediate **40** was synthesized by the reaction of 9*H*-fluoren-9-yl isocyanate (**39**) with **38** in refluxing toluene. Finally, the target compound **16** was obtained by reacting **40** in 50% THF/MeOH with Pd/C in an atmosphere of hydrogen at room temperature. To synthesize compound **17**, **41** was hydrolyzed to 2,6-bis-(2-benzyloxyethoxy)benzamide (**42**) [1], and then **17** was obtained following the same route used in the synthesis of **16**.

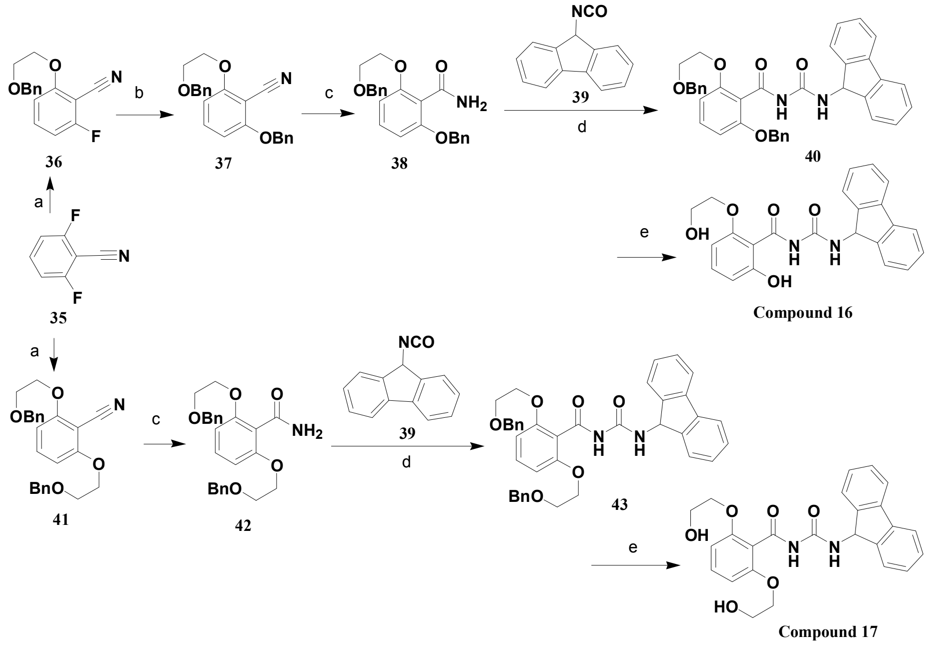

Reagents and conditions: (**a**) $C_6H_5CH_2OCH_2CH_2ONa$, DMSO, 120 °C; (**b**) sodium benzyloxide, DMSO, 120 °C; (**c**) KOH, trace water, 130 °C; (**d**) toluene, reflux; (**e**) H_2, Pd/C, THF, MeOH, r. t.

Scheme S1. The synthesis of compounds **16**, **17**.

2-(2-Benzyloxyethoxy)-6-fluorobenzonitrile (**36**). To a solution of 2-benzyloxyethanol (0.96 g) in dimethyl sulfoxide (DMSO, 5 mL), was added 60% sodium hydride (0.156 g) and the mixture was stirred in an atmosphere of nitrogen at room temperature for 3 h. To the mixture 2,6-difluorobenzonitrile (**35**, 0.47 g) was added and then the temperature was increased to 110 °C. The reaction mixture was stirred for 10 h and, after cooling, poured into 100 mL water, and extracted with dichloromethane (DCM, 40mL) three times. The organic layers were combined, dried with anhydrous sodium sulfate, and then concentrated to dryness. The residue was purified by flash column chromatography on silica gel, eluted with a mixture of ethyl acetate (EA)/PE (1:5, *v*/*v*), to afford **36** (0.77 g, 84%) as a white solid: ^{1}H-NMR ($CDCl_3$) δ: 7.54–7.44 (m, 1H), 7.44–7.25 (m, 5H), 6.80 (dt, *J* = 8.4, 3.8 Hz, 2H), 4.68 (s, 2H), 4.33–4.25 (m, 2H), 3.94–3.87 (m, 2H).

2,6-Bis-(2-benzyloxyethoxy)-benzonitrile (**41**). To a solution of 2-benzyloxyethanol (1.7 g) in DMSO (5 mL), was added 60% sodium hydride (0.45 g) and the mixture was stirred in an atmosphere of nitrogen at room temperature for 3 h. To the mixture, 2,6-difluorobenzonitrile (**35**, 0.47 g) was added and then the temperature was increased to 110 °C. The reaction mixture was stirred for 10 h and, after cooling it was poured into water (100 mL), and extracted with DCM (40mL) three times. The organic layers were combined, dried with anhydrous sodium sulfate, then concentrated to dryness. The residue was purified by flash column chromatography on silica gel, eluted with a mixture of EA/PE (1:5, *v*/*v*), to afford **41** (1.1 g, 81%) as a white solid: ^{1}H-NMR ($CDCl_3$) δ: 7.49–7.21 (m, 12H), 6.54 (d, *J* = 8.5 Hz, 1H), 4.67 (s, 2H), 4.56 (s, 2H), 4.23 (t, *J* = 4.8 Hz, 2H), 3.88 (t, *J* = 4.8 Hz, 2H), 3.81–3.71 (m, 2H), 3.66–3.53 (m, 2H).

2-Benzyloxy-6-(2-benzyloxyethoxy)-benzonitrile (**37**). To a solution of sodium benzyloxide (0.48 g) in DMSO (10 mL), was added 2-(2-benzyloxyethoxy)-6-fluorobenzonitrile (**36**, 0.45 g) with stirring and then the temperature was rapidly increased to 110 °C for 10 h. The reaction mixture was cooled and poured into water (100 mL), and extracted with DCM (40 mL) three times. The organic layers were combined, dried with anhydrous sodium sulfate, then concentrated to dryness. The residue was purified by flash column chromatography on silica gel, eluted with a mixture of EA/PE (1:5, *v*/*v*), to afford **37** (0.53 g, 89%) as a white solid: ^{1}H-NMR ($CDCl_3$) δ: 7.42–7.15 (m, 11H), 6.49 (t, *J* = 8.3 Hz, 2H), 5.12 (s, 2H), 4.59 (s, 2H), 4.16 (t, *J* = 4.8 Hz, 2H), 3.85–3.78 (m, 2H).

2-Benzyloxy-6-(2-benzyloxyethoxy)-benzamide (**38**). A mixture of **37** (0.36 g), tetrabutylammonium bromide (0.25 g) and potassium hydroxide (1 g) in benzyl alcohol (3 mL) and water (0.25 mL) was heated to 140 °C for 24 h, after which the solvent was distilled off. The residue was treated with water (50 mL), and the resulting solid collected was purified by flash column chromatography on silica gel, eluted with a mixture of EA/PE (1:4, v/v), to afford **38** (166 mg, 44%) as a white solid: ^{1}H-NMR (400 MHz, $CDCl_3$) δ: 7.44 (d, *J* = 7.3 Hz, 2H), 7.39–7.27 (m, 8H), 7.23 (t, *J* = 8.4 Hz, 1H), 6.61 (dd, *J* = 14.2, 8.4 Hz, 2H), 5.14 (s, 2H), 4.61 (s, 2H), 4.21 (t, *J* = 4.8 Hz, 2H), 3.84 (t, *J* = 4.8 Hz, 2H); EI-MS *m/z* 377.2 (M^+).

1-[2-Benzyloxy-6-(2-benzyloxyethoxy)-benzoyl]-3-(9H-fluoren-9-yl)-urea (**40**). To a solution of 9*H*-fluoren-9-yl isocyanate (**39**, 0.21g) was added **38** (0.32 g) with stirring at room temperature. The mixture was heated to 110 °C for 10 h. The solvent was evaporated under vacuum to give the crude

product that was purified by flash column chromatography on silica gel, eluted with a mixture of THF/DCM/PE (1:5:3, v/v/v), to afford **40** (347 mg, 70%) as a white solid: EI-MS *m/z* 584.2 (M^+).

1-(9H-Fluoren-9-yl)-3-[2-hydroxy-6-(2-hydroxyethoxy)-benzoyl]-urea (**16**). A mixture of **40** (0.5 g), 10% palladium on charcoal (0.2 g), methanol (30 mL) and THF (30 mL) was stirred at 40 °C for 48 h in an atmosphere of hydrogen. The solvent was evaporated under vacuum and then the residue was purified by flash column chromatography on silica gel, eluted with a mixture of THF/ DCM/PE (1:4:3, *v*/*v*/*v*), to afford **16** (246 mg, 71%) as a white solid: mp 208–213 °C; ^{1}H-NMR (acetone-d_6) δ: 12.16 (s, 1H), 10.84 (s, 1H), 8.62 (d, *J* = 7.8 Hz, 1H), 7.86 (d, *J* = 7.5 Hz, 2H), 7.71 (d, *J* = 7.4 Hz, 2H), 7.42 (dd, *J* = 30.3, 12.8, 7.3 Hz, 5H), 6.71 (d, *J* = 8.3 Hz, 1H), 6.58 (d, *J* = 8.4 Hz, 1H), 6.10 (d, *J* = 8.1 Hz, 1H), 4.43–4.36 (m, 2H), 4.29 (t, *J* = 5.0 Hz, 1H), 4.09 (dd, *J* = 9.4, 4.8 Hz, 2H); ESI-MS *m*/*z* 403.0 $[M - H]^-$; HRMS (ESI) *m/z* calcd $C_{23}H_{20}N_2O_5$ $[M - H]^-$ 403.1294, found 403.1299.

2,6-Bis-(2-benzyloxyethoxy)-benzamide (**42**). A mixture of **41** (0.81 g), tetrabutylammonium bromide (0.5 g) and potassium hydroxide (2 g) in benzyl alcohol (5 mL) and water (1 mL) was heated to 140 °C for 24 h, after which the solvent was distilled off. The residue was treated with water (50 mL), and the resulting solid collected was purified by flash column chromatography on silica gel, eluted with a mixture of EA/PE (1:4, *v*/*v*), to afford **42** (440 mg, 52%) as a white solid: ^{1}H-NMR ($CDCl_3$) δ: 7.36–7.19 (m, 11H), 6.57 (d, *J* = 8.4 Hz, 2H), 6.07 (s, 1H), 5.70 (s, 1H), 4.59 (s, 4H), 4.18 (t, *J* = 4.9 Hz, 4H), 3.82 (t, *J* = 4.9 Hz, 4H); EI-MS *m/z* 422.2 (M^+).

1-[2,6-Bis-(2-benzyloxyethoxy)benzoyl]-3-(9H-fluoren-9-yl)-urea (**43**). To a solution of 9*H*-fluoren-9-yl isocyanate (**39**, 0.19 g) was added **42** (0.32 g) with stirring at room temperature. The mixture was heated to 110 °C for 10 h. The solvent was evaporated under vacuum to give the crude product that was purified by flash column chromatography on silica gel, eluted with a mixture of THF/DCM/PE (1:5:3, *v*/*v*/*v*), to afford **43** (340 mg, 71%) as a white solid: mp 143–145 °C; ^{1}H-NMR (acetone-d_6) δ: 9.77 (s, 1H), 8.94 (d, *J* = 6.8 Hz, 1H), 7.82 (d, *J* = 7.6 Hz, 2H), 7.58 (d, *J* = 7.5 Hz, 2H), 7.41 (t, *J* = 7.5 Hz, 2H), 7.37–7.19 (m, 13H), 6.74 (d, *J* = 8.4 Hz, 2H), 6.10 (d, *J* = 8.1 Hz, 1H), 4.62 (s, 4H), 4.28–4.19 (m, 4H), 3.87–3.77 (m, 4H); EI-MS *m/z* 628.2 (M^+); 91.0 (100%); HRMS (EI) *m/z* calcd $C_{39}H_{36}N_2O_6$ (M^+) 628.2573, found 628.2571.

1-[2,6-Bis-(2-hydroxyethoxy)-benzoyl]-3-(9H-fluoren-9-yl)-urea (**17**). A mixture of **43** (0.5 g), 10% palladium on charcoal (0.2 g), methanol (30 mL) and THF (30 mL) was stirred at 40 °C for 48 h in an atmosphere of hydrogen. The solvent was evaporated under vacuum and then the residue was purified by flash column chromatography on silica gel, eluted with a mixture of THF/DCM/PE (1:4:3, *v*/*v*/*v*), to afford **17** (221 mg, 62%) as a white solid: mp 158–163 °C; ^{1}H-NMR (acetone-d_6) δ: 9.82 (s, 1H), 8.83 (d, *J* = 7.7 Hz, 1H), 7.87 (d, *J* = 7.5 Hz, 2H), 7.74 (d, J = 7.5 Hz, 2H), 7.47 (t, *J* = 7.3 Hz, 2H), 7.43–7.32 (m, 3H), 6.77 (d, *J* = 8.4 Hz, 2H), 6.12 (d, *J* = 8.0 Hz, 1H), 4.21–4.15 (m, 4H), 3.96 (t, *J* = 5.9 Hz, 2H), 3.82 (dd, *J* = 10.2, 5.4 Hz, 4H); EI-MS *m/z* 448.2 (M^+); 180.1 (100%); HRMS (EI) *m/z* calcd $C_{25}H_{24}N_2O_6$ (M^+) 448.1634, found 448.1635.

HPLC Analysis Data of Compounds 14–33

Table S1. HPLC analysis data of compounds **14**–**33**. The purities of identified compounds were essential to the conclusions drawn in the text and were determined by the same instrumentation under several different conditions given in the following table. The peak purity was checked by UV spectraoscopy.

**Equipment**	Agilent 1200 with quaternary pump and diode-array detector (DAD)				
**Column**	BECKMAN ULTRASPHERE C_{18} ODS column (4.6 × 250 mm, 5 μm particle size)				
**System Condition**	CH_3OH/H_2O, 80% (*v*/*v*) of CH_3OH gradient, flow rate: 0.5 mL·min^{-1}; the relative purity of each compound was calculated at 254 nM				
**Compound**	**Retention Time (min)**	**Relative Purity (%)**	**Compound**	**Retention Time (min)**	**Relative Purity (%)**
**14**	8.67 [a]	99.0	**15**	7.87	95.3
**16**	9.22	97.3	**17**	7.52	98.3
**18**	8.59	95.2	**26**	8.15	98.4
**19**	7.36	95.0	**27**	9.82	99.4
**20**	5.26	98.1	**28**	11.09	96.9
**21**	7.79	98.6	**29**	10.48	96.4
**22**	8.53	95.2	**30**	12.56	95.3
**23**	8.46	95.0	**31**	7.88	98.0
**24**	8.60	95.1	**32**	7.94	96.0
**25**	9.36	95.4	**33**	8.08	96.7

[a] CH_3OH/H_2O, 90% (*v*/*v*) of CH_3OH gradient, flow rate: 0.5 mL·min^{-1}, the relative purity was calculated at 254 nM.

Establishment of HCV Virus and Subgenomic Replicon Cell Lines

The HCV virus assay was constructed by using the method developed as previously described with a small modification [2,3]. In brief, the pRluc-JFH-1 plasmid was constructed as follows: based on the plasmid of pJFH-1, a gift from Apath, LLC (New York, NY, USA), a humanized Renilla luciferase reporter gene was introduced into the C-terminus of NS5A in the JFH-1 genome. The plasmid pRluc-JFH-1 was made through digestion with XbaI restriction enzyme, and used as a template for RNA transcription. The virus transcripts were prepared *in vitro* by using the Ambion MEGAscript Kit, and then 10 μg RNA was mixed with 400 mL of Huh7.5.1 cells, which were a kind gift of Jin Zhong (Institute Pasteur of Shanghai, Chinese Academy of Science, Shanghai, China), at a concentration of 1×10^7 cells·mL^{-1}. After electroporation, the Huh7.5.1 cells containing virus transcripts were seeded in a 10 cm dish. After cells were cultured for 4 days, the supernatant was collected and filtered to obtain the stock solution of virus JFH-1. To obtain virus titer, the virus stocks were diluted at a gradient of 1:10, and incubated the Huh7.5.1 cells for 48 h at 37 °C. Then the cells were harvested and the luminescence was detected as manufacturer's protocol of Renilla-Glo™ Luciferase Assay System (Promega, Beijing, China).

The plasmids containing HCV replicon genotype 1b (Con-1), 1a (1a H77) containing a luciferase reporter gene was a gift from Apath, LLC. Creation of the plasmid of genotype 2a (2a JFH-1) subgenomic replicon has been reported previously [4]. All subgenomic HCV replicons encode an HCV internal

ribosome entry site (IRES)-driven neomycin cassette and encephalomyocarditis virus-controlled coding region containing a humanized Renilla luciferase reporter gene and the nonstructural NS3, NS4A, NS4B, NS5A, and NS5B polyprotein sequences, separated by a FMDV cleavage site. All stable a linearized bicistronic construct as previously. All Huh7.5.1-based replicon cell lines were grown subgenomic replicon cells were created by electroporating Huh7.5.1 cells with RNA transcribed from in Dulbecco modified Eagle medium (DMEM) supplemented with 10% fetal bovine serum. Twenty-four hours after electroporation, replicon-containing cell clones were selected using 0.5 mg·mL^{-1} G418 (Gibco; Invitrogen, Shanghai, China). Stable replicon cell lines were selected and maintained in medium containing 0.5 mg·mL^{-1} G418.

The mutational JFH-1 recombinant virus was constructed based on the phRluc-JFH-1 plasmid. Site-directed mutagenesis was performed using a Quick Change Lighting Site-Directed Mutagenesis kit (Stratagene, Shanghai, China). The mutagenic primers were design as follows: NS5BS282T-Sense: 5′-CAGACGTTGCCGCGCCACCGGGGTGCTAACCACTAG-3′, NS5BS282T-Antisense: 5′-CTAGTG GTTAGCACCCCGGTGGCGCGGCAACGTCTG-3′. The constructs were confirmed by sequencing.

Solution Stability Assay

The most potent compound **25** was selected as a candidate for further testing by the HPLC method [5]. To a solution of **25** (1 mg) in DMSO (6 mL) was added PBS buffer (48 mg NaCl, 1.2 mg KCl, 22 mg $Na_2HPO_4·12H_2O$, 1.4 mg KH_2HPO_4, pH 7.4) at 0 °C. Then the samples were incubated at 37 °C. At defined times the solution was analyzed and samples were run on a BECKMAN ULTRASPHERE C_{18} ODS column (4.6 × 250 mm, 5 μm particle size) at 25 °C with a flow rate of 0.5 mL·min^{-1} of an isocratic eluent composed of methanol/water (70/30, *v*/*v*), and the eluted analytes were detected at 254 nm.

Table S2. The result of solution stability test of **25**.

Time (h)	Peak Area of 25	25 Content [a] (%)	Time (h)	Peak Area of 25	25 Content [a] (%)
0	83.59	100	5.0	83.77	100.21
0.5	83.42	99.79	6.0	83.80	100.24
1.0	83.70	100.13	7.0	83.88	100.34
1.5	83.40	99.76	8.0	83.84	100.30
2.0	84.20	100.72	9.0	83.00	99.29
2.5	84.47	100.05	10.0	82.66	98.88
3.0	84.16	100.67	11.0	82.28	98.43
3.5	84.37	100.93	12.0	82.46	98.65
4.0	84.21	100.74			

[a] **25** content (%) was calculated using the formula: peak area of **25** at each timepoint/the peak area of **25** at 0 h × 100%.

Pharmacokinetic Studies

The pharmacokinetic studies of compound **25** were carried out in SD rats (male SD rats (200 ± 20 g) × 3, provided by the Sun Yat-sen University Animals Center). Before administration, to a solution of **25** (4 mg) in *N*,*N*-dimethylacetamide (DMA, 100 μL) was added castor oil (900 μL), and then the solution was diluted with normal saline to 10 mL. The rats were administered at a dose of 5 mL·kg^{-1} intravenously.

After intravenous administration, blood samples (0.2 mL) were collected at designated time points (0, 5, 15, 30, 45 min, 1, 2, 3, 5, 8, 12 and 24 h) from the eye venous sinus of the rats. The blood samples were centrifuged at 14800 rpm for 10 min to obtain the plasma fraction. The plasma samples were kept frozen (−20 °C) until analysis by a liquid chromatography-tandem mass spectrometry (LC-MS/MS).

Table S3. Pharmacokinetic parameters after intravenous administration of **25** to SD rats at 5 mL·kg^{-1}.

Pharmacokinetic Parameters	25 (Intravenous Injection of 2 mg 25/kg)			
	Rat 1	Rat 2 [a]	Rat 3	Mean ± SD
[b] C_{max} (ng·mL^{-1})	13.40	N [g]	27.54	20.47 ± 9.99
[d] $AUC_{0\text{-}\infty}$ (ng·h·mL^{-1})	5.66	N [g]	5.30	5.48 ± 0.25
[f] $t_{1/2}$ (h)	5.40	N [g]	0.12	2.76 ± 3.73
[e] MRT (h)	2.42	N [g]	0.21	1.3 ± 1.6
	**1 (intravenous injection of 2mg 25/kg)**			
	**Rat 1**	**Rat 2 [a]**	**Rat 3**	**Mean ± SD**
[b] C_{max} (ng·mL^{-1})	698.12	52.28	1110.90	904.5 ± 291.9
[c] T_{max} (h)	0.083	0.083	0.083	0.083 ± 0
[d] $AUC_{0\text{-}\infty}$ (ng·h·mL^{-1})	335.12	78.78	323.69	329.4 ± 8.08
[f] $t_{1/2}$ (h)	1.84	1.10	1.21	1.53 ± 0.45
[e] MRT (h)	0.94	1.98	0.55	0.75 ± 0.28

[a] Rat 2: parts of the sample of **25** was injected into the subcutaneous tissues which lead to the uncertainty of the plasma concentration, so this rat was not incorporated into the calculation of pharmacokinetic parameters; [b] C_{max}: maximum plasma concentration taken directly from measured values; [c] T_{max}: time to reach C_{max}; [d] $AUC_{0\text{-}\infty}$: integrated area under plasma concentration *vs.* time curve from time 0 to time infinity; [e] MRT: mean residence time; [f] $t_{1/2}$: half-life. [g] N: parts of the data below the LLOQ, that result in the uncertainty of the pharmacokinetic parameters.

hERG Cardiac Toxicity Assay of Compound 25

Cell Culture and Cell Requirements

A CHO cell line stably transfected with hERG cDNA and expressing hERG channels was used for the study. The cells were cultured in medium containing the following components: Ham's F12, 10% (*v*/*v*) heat inactivated FBS, 100 μg·mL^{-1} hygromycin B and 100 μg·mL^{-1} geneticin. The cells used in QPatch study must meet following criteria: under microscopy examination, the majority of cells in suspension should be single and isolated; their viability should be greater than 95%, with only a few debris and cell clumps (which may clog the holes in QPlate during whole-cell clamp recording); cell density should be ranged within 3–8 × 10^{6} cells·mL^{-1} in the final suspension before applying to the QPatch stir chamber. After leaving CO_2 incubator, cells are maintained in serum-free medium buffered HEPES. Cells in such condition can be used for recording only for four hours after harvesting.

Recording System

Whole-cell recordings were performed using automated QPatch (Sophion, Ballerup, Denmark). The cells were voltage clamped at a holding potential of −80 mV. The hERG current was activated by

depolarizing at +20 mV for 5 s, after which the current was taken back to −50 mV for 5 s to remove the inactivation and observe the deactivating tail current. The maximum amount of tail current size was used to determine hERG current amplitude. The composition of the solutions used for the electrophysiological recordings were described in Table S4.

Table S4. Composition of internal and external solutions used in hERG QPatch studies [a].

Reagents	External Solution (mM)	Internal Solution (mM)
**$CaCl_2$**	2	5.374
**$MgCl_2$**	1	1.75
**KCl**	4	120
**NaCl**	145	-
**Glucose**	10	-
**HEPES**	10	10
**EGTA**	-	5
**Na-ATP**	-	4
**pH**	7.4 (adjusted with NaOH), osmolarity ~305 mOsm	7.25 (adjusted with KOH), osmolarity ~290 mOsm

[a] solutions recommended by Sophion.

Automated QPatch Procedures

After achieving break-in (whole-cell) configuration, the cells were recorded for 120 s to assess current stability. The voltage protocol described above was then applied to the cells every 20 s throughout the whole procedure. Only stable cells with recording parameters above threshold were allowed to enter the drug addition procedure.

External solution containing 0.2% DMSO (vehicle) was applied to the cells to establish the baseline. After allowing the current to stabilize for 3 min, compound **25** (20 mM stocked in DMSO) was applied. The solution of **25** (40, 8, 1.6, 0.32, 0.064 and 0.0128 μM, first diluted in DMSO, and then serial-diluted in external solution to the final μM ranges before QPatch test. Final DMSO concentration was 0.2%) was added and the cells were kept in the test solution until the compound's effect reached a steady state or for a maximum of 3 min. Washout with external solution might be performed until the recovery of the current reached a steady state. Positive control cisapride (dosing started at 3 μM) is used in the experiments to test the same batch of cells used for test compounds to ensure the normal response and the good quality of the cells. The method described above was performed according to [6–8].

Supporting References

1. Wellinga, K.; Mulder, R.; van Daalen, J.J. Synthesis and laboratory evaluation of 1-(2,6-disubstituted benzoyl)-3-phenylureas, a new class of insecticides. II. Influence of the acyl moiety on insecticidal activity. *J. Agric. Food Chem.* **1973**, *21*, 993–998.
2. Han, Q.; Xu, C.; Wu, C.; Zhu, W.; Yang, R.; Chen, X. Compensatory mutations in NS3 and NS5A proteins enhance the virus production capability of hepatitis C reporter virus. *Virus Res.* **2009**, *145*, 63–73.

3. Wu, Y.; Liao, Q.; Yang, R.; Chen, X.; Chen, X. A novel luciferase and GFP dual reporter virus for rapid and convenient evaluation of hepatitis C virus replication. *Virus Res*. **2011**, *155*, 406–414.
4. Hao, W.; Herlihy, K.; Zhang, N.; Fuhrman, S.; Doan, C.; Patick, A.; Duggal, R. Development of a novel dicistronic reporter-selectable hepatitis C virus replicon suitable for high-throughput inhibitor screening. *Antimicrob. Agents Chemother*. **2007**, *51*, 95–102.
5. Miao, Z.; Sheng, C.; Zhang, W.; Ji, H.; Zhang, J.; Shao, L.; You, L.; Zhang, M.; Yao, J.; Che, X. New homocamptothecins: Synthesis, antitumor activity, and molecular modeling. *Bioorg. Med. Chem*. **2008**, *16*, 1493–1510.
6. Kutchinsky, J.; Friis, S.; Asmild, M.; Taboryski, R.; Pedersen, S.; Vestergaard, R.K.; Jacobsen, R.B.; Krzywkowski, K.; Schrøder, R.L.; Ljungstrøm, T.; *et al*. Characterization of potassium channel modulators with QPatch automated patch-clamp technology: System characteristics and performance. *Assay Drug Dev. Technol.* **2003**, *1*, 685–693.
7. Zhou, P.; Babcock, J.; Liu, L.; Li, M.; Gao, Z. Activation of human ether-a-go-go related gene (hERG) potassium channels by small molecules. *Acta Pharmacol. Sin*. **2011**, *32*, 781–788.
8. Zhou, Z.; Gong, Q.; Ye, B.; Fan, Z.; Makielski, J.C.; Robertson, G.A.; January, C.T. Properties of HERG channels stably expressed in HEK 293 cells studied at physiological temperature. *Biophys. J.* **1998**, *74*, 230–241.
